# XGBoost, a Machine Learning Method, Predicts Neurological Recovery in Patients with Cervical Spinal Cord Injury

**DOI:** 10.1089/neur.2020.0009

**Published:** 2020-07-23

**Authors:** Tomoo Inoue, Daisuke Ichikawa, Taro Ueno, Maxwell Cheong, Takashi Inoue, William D. Whetstone, Toshiki Endo, Kuniyasu Nizuma, Teiji Tominaga

**Affiliations:** ^1^ Department of Neurosurgery, National Health Organization Sendai Medical Center, Sendai, Miyagi, Japan.; ^2^ SUSMED, Inc., Tokyo, Japan.; ^3^ Department of Radiology, Stanford University School of Medicine, Palo Alto, California, USA.; ^4^ Department of Emergency Medicine, University of California, San Francisco, San Francisco, California, USA.; ^5^ Department of Neurosurgery, Tohoku University Graduate School of Medicine, Sendai, Miyagi, Japan.; ^6^ Department of Neurosurgical Engineering and Translational Neuroscience, Graduate School of Biomedical Engineering, Tohoku University, Sendai, Miyagi, Japan.; ^7^ Department of Neurosurgical Engineering and Translational Neuroscience, Tohoku University Graduate School of Medicine, Sendai, Miyagi, Japan.

**Keywords:** cervical spinal cord injury, extreme gradient boosting, machine learning, receiver operating curve

## Abstract

The accurate prediction of neurological outcomes in patients with cervical spinal cord injury (SCI) is difficult because of heterogeneity in patient characteristics, treatment strategies, and radiographic findings. Although machine learning algorithms may increase the accuracy of outcome predictions in various fields, limited information is available on their efficacy in the management of SCI. We analyzed data from 165 patients with cervical SCI, and extracted important factors for predicting prognoses. Extreme gradient boosting (XGBoost) as a machine learning model was applied to assess the reliability of a machine learning algorithm to predict neurological outcomes compared with that of conventional methodology, such as a logistic regression or decision tree. We used regularly obtainable data as predictors, such as demographics, magnetic resonance variables, and treatment strategies. Predictive tools, including XGBoost, a logistic regression, and a decision tree, were applied to predict neurological improvements in the functional motor status (ASIA [American Spinal Injury Association] Impairment Scale [AIS] D and E) 6 months after injury. We evaluated predictive performance, including accuracy and the area under the receiver operating characteristic curve (AUC).

Regarding predictions of neurological improvements in patients with cervical SCI, XGBoost had the highest accuracy (81.1%), followed by the logistic regression (80.6%) and the decision tree (78.8%). Regarding AUC, the logistic regression showed 0.877, followed by XGBoost (0.867) and the decision tree (0.753). XGBoost reliably predicted neurological alterations in patients with cervical SCI. The utilization of predictive machine learning algorithms may enhance personalized management choices through pre-treatment categorization of patients.

## Introduction

Cervical spinal cord injury (SCI) leads to poor neurological disabilities that are associated with a deteriorated quality of life and higher rate of unemployment.^1,2^ In the hospital, these patients are assessed neurologically or radiographically to ascertain their neurological impairments and select adequate treatment strategies. Accurate predictions of neurological outcomes are important for effectively maximizing limited medical resources. Therefore, a dependable outcome prediction model is crucial for estimating recovery following SCI and assisting family or informal caregivers in providing personalized care.

Compared with conventional statistical models, such as a logistic regression analysis, machine learning prediction models detect non-linear interactions among prognostic factors.^3^ Previous studies reported that machine learning models were useful for predicting the outcomes of various clinical entities, such as traumatic brain injury,^4^ congestive heart failure,^5^ sepsis,^6^ asthma,^7^ and chronic obstructive pulmonary disease,^8^ or of intensive care.^9^ Some prognostic models have been validated to optimize management. Also, the requirement for effective outcome prediction in patients with SCI has increased numerous research studies evaluating the efficacy of machine learning algorithms for this cohorts.^10–14^

Among different machine learning systems, extreme gradient boosting (XGBoost) is widely used to accomplish state-of-the-art analyses in diverse fields with good accuracy or area under the receiver operating characteristic curve (AUC).^15,16^ XGBoost, a decision-tree-based ensemble machine learning algorithm with a gradient boosting framework, was developed by Chen and Guestrin.^17^ It has since been used in traffic census and the field of energy consumption.^18,19^ This is the first study to examine the efficacy of XGBoost for predicting neurological outcomes in patients with cervical SCI. The study's purpose was not to improve prognostic models based on a large number of predictor variables, but to innovate machine learning models based on XGBoost using clinical information regularly obtained from patients with SCI on admission.

## Methods

### Study design, ethical approval, and setting

We retrospectively identified patients with a principal diagnosis of SCI (code S14) according to the *International Classification of Diseases, 10th Revision, Clinical Modifications*, from diagnosis codes on admission. We also included patients with SCI who were initially diagnosed at a local hospital and then transferred to our hospital (National Health Organization Sendai Medical Center) for intensive care. To decrease possible confounding factors due to different surgical strategies, we excluded all patients who underwent surgery in other hospitals. We also excluded patients with a neurological disease or deficits (e.g., Parkinson's disease or stroke) prior to injury. Research procedures were approved by the Institutional Review Board of Sendai Medical Center, which exempted us from the need to obtain consent from individual participants.

### Data collection

Basic data were attained from the Sendai Medical Center's Department of Neurosurgery database, and were cross-referenced with trauma records and searchable terms in electronic medical records. Patient demographic data were routinely recorded in our department during the study period, and a data dictionary was utilized to assure consistent data sharing across sites. After collection, all data were evaluated for completeness and accuracy, and then anonymized before investigation. Data were acquired on age, sex, previous medical history, neurological severity, magnetic resonance imaging (MRI) findings, and surgical procedures. Following an intensive review of all variables in database files, we selected 44 basic variables and categorized them into independent categories: demographics and neurological status (8 features), mechanisms of injury (l feature), treatment strategies (7 features), radiographic information (14 features), and concomitant degenerative spine disease (7 features) (Table 1). To reduce selection bias, the authors responsible for chart reviews were blinded to neurological outcomes.

**Table 1.** Patient Characteristics

Forty-four predictors	
Demographics and neurological status (8)	
Age (years), mean (SD)	65.3 (15.4)
Sex (*n*)	132 males, 33 females
Height, mean (SD)	164.2 (10)
Body weight, mean (SD)	64.1 (14.0)
Body mass index, mean (SD)	24.1 (7.6)
Body surface area, mean (SD)	1.70 (0.24)
American Spinal Injury Association Impairment Scale (AIS) (*n*)	AIS A = 15, B = 38, C = 66, D = 44, E = 3
Charlson Comorbidity Index, median (IQR)	0 (0 - 1)
Mechanism of injury (8)	
Slip (*n*)	78
Fall (*n*)	36
Loss of consciousness (*n*)	10
Motor vehicle collision (*n*)	22
Bicycle (*n*)	11
Sports (*n*)	8
Spinal cord injury after alcohol consumption (*n*)	37
Spinal cord injury with traumatic brain injury (*n*)	137
Therapeutic strategies for spinal cord injury (7)	
Surgical timing, median (days) (IQR)	2 (1 - 7)
Conservative therapy (*n*)	36
Anterior cervical discectomy and fusion (*n*)	43
Posterior fixation (*n*)	6
Laminoplasty (n)	82
Halo-vest stabilization (*n*)	3
Methylprednisolone use (*n*)	39
Radiographic information (14)	
Brain and Spinal Cord Injury Center score (BASIC) (*n*)	BASIC 0 = 21, 1 = 61, 2 = 46, 3 = 24, 4 = 14
Longest measurements of T2 hyperintensity on the sagittal plane (mm), mean (SD)	14.4 (13.7)
Sagittal grading, median (IQR)	2 (2 - 2.25)
Subaxial Injury and Classification system, median (IQR)	6 (5 - 6)
Maximum canal compromise, mean (SD)	75.9 (3.04)
Maximum spinal cord compression, mean (SD)	79.9 (4.56)
Signal intensity at the narrowest level on T1-weighted images, mean (SD)	293.0 (69.3)
Signal intensity at the narrowest level on T2-weighted images, mean (SD)	389.0 (152.2)
Signal intensity at the C7-T1 disc levels on T1-weighted images, mean (SD)	276.7 (66.8)
Signal intensity at the C7-T1 disc levels on T2-weighted images, mean (SD)	268.9 (98.1)
Signal intensity ratio on T1-weighted images, mean (SD)	1.07 (0.15)
Signal intensity ratio on T2-weighted images, mean (SD)	1.40 (0.37)
Cervical alignment (*n*)	Lordotic (73), reverse S-shape (37), straight (27), kyphosis (15), dislocation (8)
High cervical (*n*)	36
Concomitant degenerative spine diseases (7)	
Cervical spondylosis	144
Ossification of the posterior longitudinal ligament	52
Cervical disc herniation	64
Osteophyte	115
Ossification of the yellow ligament	2
Ankylosing spondylitis	20
Atlantoaxial dislocation	6

IQR, interquartile range; SD, standard deviation.

All patients were evaluated by our multi-disciplinary team immediately after being transferred to our hospital; this team consisted of board-certified neurosurgeons, emergency physicians, and radiologists. All patients were evaluated using the ASIA (American Spinal Injury Association) Impairment Scale (AIS). A complete radiological evaluation, including standard radiographs and computed tomography, was performed for each patient to assess the degree of compression and injury to the spinal cord. Patients also underwent MRI within 24 h of the traumatic event, including T1- and T2-weighted imaging (WI) of the cervical spine in both axial and sagittal views. MRI was performed using the 1.5 Tesla system (Magnetom Avanto, Siemens Medical Solutions, Erlangen, Germany). Previous comorbidities were recorded based on patient self-reports during hospitalization or medical histories in electronic records, using the Charlson Comorbidity Index (CCI) as a measure of clinical importance.^20,21^

### Criteria for surgical decompression

Indications for surgical decompression were the existence of surgically amenable cervical spinal cord compression due to cervical spondylosis, ossification of the posterior longitudinal ligament, or cervical disc herniation, which were considered to be responsible for neurological impairments. The timing of surgical decompression depended on the patient's condition and any comorbidities, radiological evaluation, and optimum preparation of surgical suites. The surgical approach selected was based on the finding of cord compression and the surgeon's preference. Further, in our institution, we do not regard age as an exclusion criterion for early surgery, and, thus, we often perform this surgery independent of age. Post-operatively, all patients underwent early rehabilitation consisting of physical therapy that was immediately initiated after cardiopulmonary stabilization.

### Statistical analysis

Two attending neurosurgeons performed consensus MRI ratings for all metrics while blinded to neurological outcomes. We applied the following axial scoring system, known as the Brain and Spinal Injury Center (BASIC) score^22^: grade 0, no intramedullary signal abnormality; grade 1, T2 hyperintensity confined to the gray matter; grade 2, intramedullary T2 hyperintensity extending beyond possible spinal gray matter margins to include the white matter, but not containing the whole transverse extent of the spinal cord; grade 3, intramedullary T2 hyperintensity containing the entire axial level of the spinal cord; and grade 4, a grade 3 injury plus a distinct T2 hypointense area, consistent with macroscopic intramedullary hemorrhage.^23,24^ The longitudinal extent of T2 hyperintensity (in millimeters) was evaluated based on the National Institutes of Health/National Institutes of Neurological Disorders and Stroke SCI common data elements version 1.0.^22,25,26^ Sagittal grading was evaluated based on previous studies^22,25^: grade 1, no spinal cord abnormal intensity; grade 2, one-level T2 hyperintensity; grade 3, more than a two-level T2 signal hyperintensity; and grade 4, T2 signal hyperintensity with lesions of hypointensity indicating hemorrhage.

We also evaluated the Subaxial Injury and Classification (SLIC) system, which was scored based on the importance of three factors associated with the treatment of cervical injuries: morphology, neurological status, and the integrity of the discoligamentous complex.^27^ Maximum canal compromise (MCC) and maximum spinal cord compression (MSCC) were evaluated on mid-sagittal images by differentiating the anteroposterior diameter of the canal (on sagittal T1WI for MCC) and that of the spinal cord (on sagittal T2WI for MSCC) by means of the canal or spinal cord above and below as reported previously.^25,28^ The signal intensity ratio (SIR) at the narrowest level of the spinal cord on sagittal views of T1WI and T2WI was measured, and regions of interest (ROIs) were acquired by 0.05 cm^2^. Normal cord SIs at the C7-T1 disc level were acquired, and ROIs were acquired by 0.3 cm^2^. SIRs between regions of 0.05 and 0.3 cm^2^ were calculated. SIRs on T1WI and T2WI were calculated using the following equation^29^:

$$SIR=\left(SI\left[0.05cm^{2}\right]\right)∕\left(\text{SI of the sagittal normal cord between the C7 and T1 disc levels}\left[0.3cm^{2}\right]\right).$$

Radiographs were also obtained using normal radiographic methods in which the tube was positioned on the C5 disc. The radiographic film cassette was 150 cm from the tube.^30^ Study participants were categorized into four groups based on differences in alignment in the upright position: lordotic, straight type, kyphotic, S-shape curvature, and dislocation.

### Machine learning

By using machine learning algorithms, we built prediction models for neurological improvements evaluated 6 months after injury based on the AIS. We dichotomized the scale as follows: AIS D or E as 1 and AIS A, B, or C as 0.

As predictors for prediction models, we included routinely obtained clinical data on admission, such as age, sex, severity of neurological impairments based on the AIS, and several MRI findings. All predictors are shown in Table 1.

We built multiple prediction models using XGBoost and logistic regressions and evaluated them by 8-fold cross validation. XGBoost is an ensemble learning algorithm and applies decision trees as base learners.^17–19^ A logistic regression analysis is a well-known method for building clinical prediction models.^31,32^ It is a type of generalized linear model and features are additively and linearly built into the model.

### Evaluation and variable importance

To evaluate prediction models, we drew a receiver operating characteristic curve (ROC curve) and calculated the area under the ROC curve (AUC). We made a confusion matrix and calculated accuracy, the true-positive rate, and the false-positive rate, as follows:

**Table:** Confusion Matrix

	Actual: Positive (AIS D/E)	Actual: Negative (AIS A/B/C)
Prediction: Positive	TP (true-positive rate)	FP (false-positive rate)
Prediction: Negative	FN (false-negative rate)	TN (true-negative rate)

$$Accuracy\left(\%\right)=\frac{TP+TN}{TP+TN+FP+FN}$$

We acquired the variable importance of each predictor from the XGBoost model. Variable importance indicates the usefulness of each predictor for the prediction model and was calculated for a single decision tree based on the amount that each attribute split-point improved the performance measure, weighted by the number of observations for which the node was responsible.^3^

## Results

### Baseline characteristics of participants

During the study period, 165 patients (132 men and 33 women) aged 16 to 93 years (median, 68 years) and diagnosed with cervical SCI were examined. Key demographic, clinical, and outcome parameters in the present study are summarized in Table 1.

### Comparison with other prediction models

**FIG. 1.**
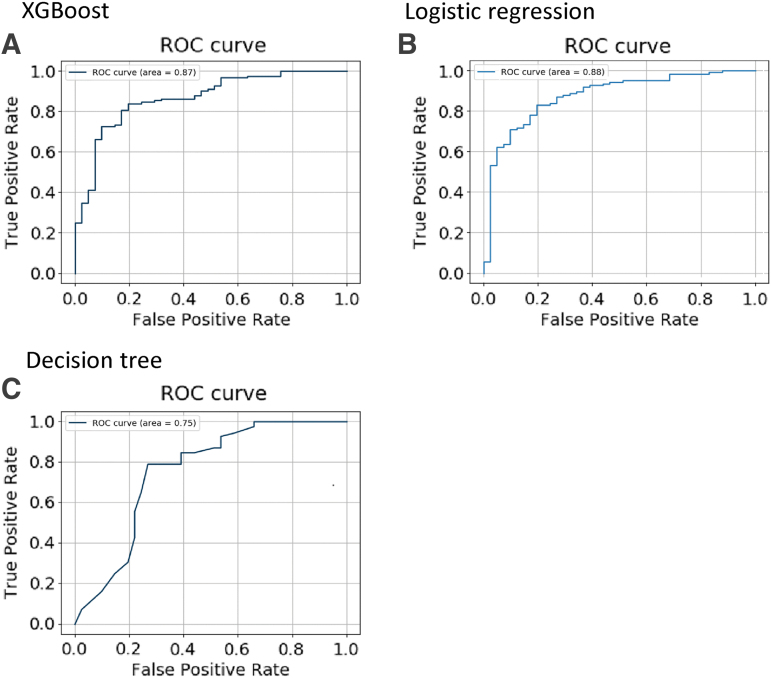
Receiver operating characteristic curves for models with all algorithms as inputs.

We performed three algorithms utilizing the training set to obtain better predictors by restoring the parameters of each algorithm and adjusted the predictors based on the validation set. Table 2 shows the prediction capability (accuracy and AUC) of each algorithm using the optimal features subset. As shown in Figure 1, XGBoost and the logistic regression predicted neurological recovery with an AUC greater than 0.800, and XGBoost showed the best performance for outcome predictions. It had the highest accuracy, 81.1%, followed by the logistic regression (80.6%) and decision tree (78.8%). Regarding AUC, the logistic regression showed 0.877, followed by XGBoost (0.867) and the decision tree (0.753).

**Table 2.** Confusion Matrix for XGBoost, a Logistic Regression, and a Decision Tree

	XGBoost			Logistic regression			Decision tree		
	Actual positive (AIS D/E)	Actual negative (AIS A/B/C)	Total	Actual positive (AIS D/E)	Actual negative (AISA/B/C)	Total	Actual positive (AIS D/E)	Actual negative (AIS A/B/C)	Total
Prediction: positive	115 (TP)	23 (FP)	138	101 (TP)	10 (FP)	111	110 (TP)	17 (FP)	127
Prediction: negative	9 (FN)	18 (TN)	27	23 (FN)	31 (TN)	54	14 (FN)	24 (TN)	38
Total	124	41	165	124	41	165	124	41	165

AIS, American Spinal Injury Association Impairment Scale; FN, false-negative; FP, false-positive; TN, true-negative; TP, true-positive.

### Variable importance

**FIG. 2.**
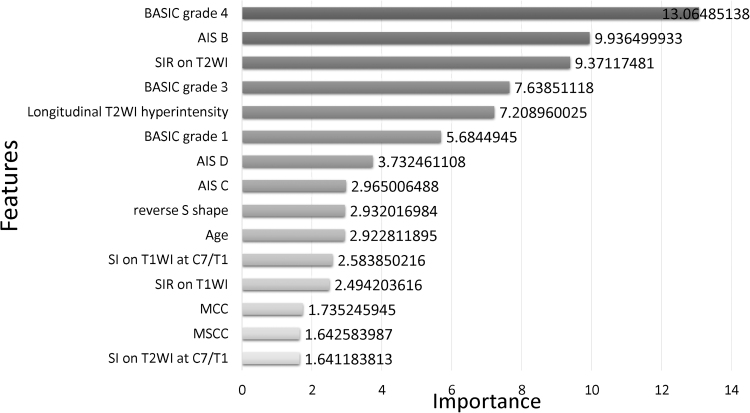
Feature importance of factors predicting neurological improvements in XGBoost. The top 14 features of importance are shown from high to low.

XGBoost calculates feature importance via the Gini index. To clarify the importance of each predictor, Figure 2 recapitulates the final 15 most significant variables of XGBoost after the exclusion of 30 unimportant predictors. The top 15 predictors, as scored by XGBoost, are as follows:

1. Demographics and neurological status (4): age, AIS B, C, and D
2. Mechanisms of injury (0)
3. Treatment strategies (0)
4. Radiographic information (11): BASIC 1, 3, and 4, longest measurements of T2 hyperintensity on the sagittal plane; MCC, MSCC, SIR at the narrowest level on T1WI and T2WI; SI at C7-T1 on T1WI and T2WI; and the reverse S-shape alignment
5. Concomitant degenerative spine disease (0)

**FIG. 3.**
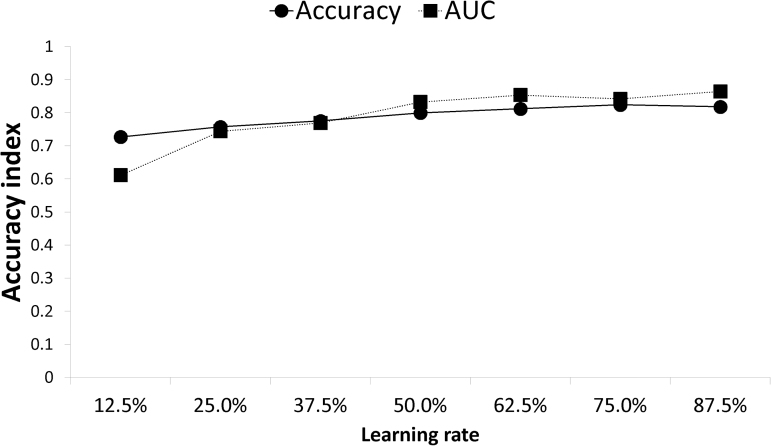
Relationship between accuracy and the number of evaluators in XGBoost.

In this model, the most important predictive variable was a BASIC score of 4, followed by AIS B, SIR on T2WI, and a BASIC score of 3 as the most significant characteristics for neurological improvements. In comparison with the two other traditional machine learning models, the accuracy rate of XGBoost was satisfactory, and the XGBoost model showed good outcomes on the ROC curve. SIR on T1WI is the most important feature of a logistic regression model, but it contributed only slightly to XGBoost. Figure 3 shows the relationship between accuracy and the number of evaluators in XGBoost. With increases in the number of predictors, computational precision is beyond 0.800 and stable at approximately 0.864, which indicates that the XGBoost algorithm accomplished sustainable predictions. When the estimates surpass 0.875, the calculation exactness of XGBoost declines, suggesting a small number of estimators.

The confusion matrix that resulted from the prediction of neurological improvements based on XGBoost, the logistic regression, and the decision tree corresponded to true-positive rates of 0.833, 0.909, and 0.866, respectively, and false-positive rates of 0.560, 0.243, and 0.415, respectively (Table 2).

## Discussion

### Results of the present study

Recovery from cervical SCI involves important tasks and significant choices to effectively utilize limited medical resources. The application of clinical information may enhance the accuracy of outcome predictions in patients with SCI. In the present study in 165 patients with cervical SCI, we applied XGBoost to regularly obtained clinical data and achieved greater prediction accuracy than that using two other models: an ordinal logistic regression analysis and a decision tree. Predictive variables analyzed according to statistical calculations for uncomplicatedness utilize simple limited fundamental variables, with measurements being approximately smoothed. Based on this algorithm, clinicians may individualize the management of patients with SCI based on their neurological alterations, which may efficiently reduce medical expenses and establish predictions for personalized neurotherapeutics for these patients.

The results of statistical analyses indicate that the majority of the selected features were instructive for predicting neurological improvements. Based on the XGBoost model, severe axial damage with a BASIC score of 4, AIS B, SIR on the T2WI scale, and BASIC grade 3 were strong predictors, in this order of importance. Previous studies reported that the assessment of intramedullary T2 signal abnormalities in the axial plane according to BASIC scores^22^ or longitudinal lesion lengths^33^ may provide important information for predicting neurological outcomes in patients with SCI. In the present study, we demonstrated that among various features, a BASIC score of 4 was the most predictive of the outcome. Talbott and colleagues reported that all patients with a BASIC score of 4 were discharged with an unchanged AIS A.^22^ In our series, 14 patients received a BASIC score of 4: 9 were AIS A and 5 were ASIA B, and none reached AIS D or E. On the other hand, among 23 patients with a BASIC score of 3, 10 (43.5 %) reached AIS D or E. XGBoost may successfully provide surgeons with selection strategies based on the potential for neurological improvements in patients with SCI.

Various factors contributed to the advancement of XGBoost in prediction modeling. State-of-art machine learning algorithms, such as XGBoost, have the capacity to analyze complex non-linear relationships among various clinical factors.^34,35^ Further, XGBoost may subjectively evaluate a number of clinical prognostic factors that were previously investigated.^22,36–38^ In addition, although overfitting is a common limitation in refined non-linear machine learning algorithms, XGBoost supervises machine learning problems by parallel computing, regularization, cross validation, flexibility, or availability.^16,39,40^

### Comparison with previous research

DeVries and associates previously reported that no clinically significant differences were observed between the use of unsupervised machine learning with complete admission neurological information and established standards.^10^ They showed the inherent weakness of applying AUC to imbalanced data sets and outlined a new strategy to evaluate performance.^10^ Tay and co-workers proposed a machine learning technique for the diagnosis of SCI using diffusion tensor imaging.^11^ They developed a classification scheme for identifying healthy individuals and patients, and reported normal case specificity of 0.912 and abnormal case sensitivity of 0.952.^11^ Khan and colleagues speculated that machine learning-based predictions will become a crucial algorithm in treatment modalities employed by spinal surgeons.^12^ Machine learning has potential and future applicability in multiple clinically significant domains due to its novelty and computational power in the area of SCI.^12^ Schwartz and associates reported that machine learning may effectively harness the value of electronic medical records in spine surgery because of developments in algorithms in reading images and in the ability to predict clinical outcomes of patients.^13^ McCoy and co-workers stated that targeted convolutional neural network training in SCI improves algorithm performance for this cohort and provides clinically relevant metrics of cord injury.^41^

In future studies, we aim to address the following. First, because XGBoost is a method for optimization, an efficient approach needs to be developed to achieve superior prognostic validity. In addition, we need to confirm whether other developed categorical procedures have a superior prognostic ability to provide clinicians with further state-of-the-art decision-making modalities. More patient information needs to be collected from medical record resources for analyses of the generalization capability of the present algorithm. Significant and precise outcome predictions may be performed when various machine learning systems, including XGBoost, are utilized in diverse clinical areas.

The present study may have been limited by the general validation of prognostic models for other data sets. Our prognostic model was produced using data from a single institution, and this needs to be considered if the model is employed in other hospitals with different treatment procedures or patient backgrounds, because it may lead to invalid prognostic importance. Further, we omitted patients with missing data, therefore logistic regression might be better than XGBoost with respect to AUC. Nusinovici and colleagues have stated that low dimensional settings include low number of events and predictors, so in such settings, logistic regression yields performance as good as machine learning models.^42^

To overcome this situation, we need to perform multi-center trials to obtain more data sets. Although further examinations on novel acute neurochemical biomarkers, such as S100ß, neurofilaments, and glial fibrillary acidic protein, may increase accuracy, ^43^ this method may not be practical in selection strategies for neurointensive treatments because of low specificity or potential cross contamination by hemolysis. However, analyzed and non-analyzed populations generally have similar backgrounds, clinical findings, or neurological outcomes. In addition, treatment strategies for patients with SCI frequently rely on a surgeon's preference. The present results showed that surgical timing did not play a major role in predicting neurological alterations assessed by AIS. However, because the importance of surgical intervention has been widely reported in many cases,^44–49^ this result may have been due to the retrospective nature of the present study affecting surgical timing, which was a possible bias. The present study was also restricted by its dependence on neurological outcomes based on AIS; this was due to the effect of selection bias that reduced the statistical power. Further studies that include other outcome measures, such as functional, psychosocial, sexual health, autonomic, bowel and bladder, and pain tools, are needed.^10,50^

In conclusion, the present study results revealed the potential of XGBoost to predict neurological alterations prior to treatments. By considering neurological recovery in patients with SCI before surgery, we may provide appropriate individualized management strategies for these patients. The present results are promising and represent the primary step for improving prognostic models that may be applied to the management of SCI in patients with a strong possibility of neurological recovery.^33^

## Abbreviations Used

AIS: ASIA Impairment Scale
ASIA: American Spinal Injury Association
AUC: area under the receiver operating characteristic curve
BASIC: Brain and Spinal Injury Center
CCI: Charlson Comorbidity Index
MCC: maximum canal compromise
MRI: magnetic resonance imaging
MSCC: maximum spinal cord compression
ROC curve: receiver operating characteristic curve
SCI: spinal cord injury
SIR: signal intensity ratio
SLIC: Subaxial Injury and Classification
WI: weighted imaging
XGBoost: extreme gradient boosting
